# TOB1 attenuates IRF3-directed antiviral responses by recruiting HDAC8 to specifically suppress IFN-β expression

**DOI:** 10.1038/s42003-022-03911-x

**Published:** 2022-09-09

**Authors:** Zhongxia Yu, Lijuan Wang, Jing Zhao, Hui Song, Chunyuan Zhao, Wei Zhao, Mutian Jia

**Affiliations:** 1 Department of Immunology, School of Basic Medical Science, Cheeloo College of Medicine, Shandong University, 250012 Jinan, Shandong China; 2 Pathology Tissue Bank, Qilu Hospital, Cheeloo College of Medicine, Shandong University, 250012 Jinan, Shandong China; 3 Department of Cell Biology, School of Basic Medical Science, Cheeloo College of Medicine, Shandong University, 250012 Jinan, Shandong China

**Keywords:** Infection, Innate immunity, Molecular biology

## Abstract

Interferon regulatory factor 3 (IRF3) is a key transcription factor required for the secretion of type I interferons (IFN-α/β) and initiation of antiviral immune response. However, the negative feedback regulator of IRF3-directed antiviral response remains unknown. In this study, we demonstrated that viral infection induced the interaction of the transducer of ERBB2.1 (TOB1) with IRF3, which bound to the promoter region of *Ifnb1* in macrophages. TOB1 inhibited *Ifnb1* transcription by disrupting IRF3 binding and recruiting histone deacetylase 8 (HDAC8) to the *Ifnb1* promoter region. Consequently, TOB1 attenuated IRF3-directed IFN-β expression in virus-infected macrophages. *Tob1* deficiency enhanced antiviral response and suppressed viral replication in vivo. Thus, we identified TOB1 as a feedback inhibitor of host antiviral innate immune response and revealed a mechanism underlying viral immune escape.

## Abstract (web summary)

TOB1 is identified as an interferon regulatory factor 3 (IRF3) binding partner that operates as a negative feedback inhibitor of IFNβ in toll-like receptor and cytosolic nucleic acid receptor sensing pathways.

## Introduction

Secretion of type I interferon (IFN-α/β) is crucial for the host immune response against invading viruses^1^. Multiple pattern recognition receptors (PRRs), including Toll-like receptors (TLRs), retinoic acid-inducible gene-I (RIG-I)-like receptors (RLRs), and cyclic GMP-AMP synthase (cGAS), detect viral infection, trigger the activation of interferon regulatory factor 3 (IRF3), and subsequently initiate the type I IFN expression^2^. Type I IFNs bind to the IFN-α/β receptor (IFNAR), activate the JAK/STAT pathway, and promote the expression of numerous antiviral genes known as interferon-stimulated genes (ISGs) to suppress viral replication^3^. Therefore, IRF3-directed transcription of type I IFNs is vital for the host to eradicate the invading viruses. Viruses have evolved elaborate strategies to circumvent IFN-mediated responses and disrupt the innate immune system. However, the mechanisms by which viruses block IRF3 activation and escape the immune system are not fully understood.

The transducer of ERBB2.1 (TOB1) is a member of the TOB/B cell translocation gene (BTG) family^4,5^. These family members are characterized by a conserved N-terminal TOB/BTG homology domain that can bind to transcription factors. BTG/TOB family members regulate cell cycle progression in a variety of cell types^4,5^. TOB1 is expressed in most cell types and helps suppress tumor development^6^. In the zebrafish embryo, TOB1 controls dorsal development by interacting with mothers against decapentaplegic homolog 3 (SMAD3) and by inhibiting β-catenin transcriptional activity^7^. In osteoblasts, TOB1 associates with SMADs to regulate osteoblast proliferation and differentiation^8^. In hepatocytes, TOB1 modulates cyclin-dependent kinase activity, inhibits the transcription of critical cell cycle components, and controls regeneration^9^. In T lymphocytes, TOB1 associates with Smad2 and Smad4 to enhance Smad4 DNA-binding and Smad-dependent transcription, and thereby it negatively regulates interleukin (IL)-2 transcription and T-cell proliferation^10^. *Tob1* deficiency in T cells promotes an aberrant T-cell immune response and drives the development of experimental autoimmune encephalomyelitis^11^. Although TOB1 plays an important role in T cells, its function in innate immune cells remains unclear.

The crystal structure revealed that IRF3 has marked structural and surface electrostatic potential similarities to the MH2 domain of the Smad protein family^12,13^. TOB1 interacts with various Smads to suppress their transcriptional activity, suggesting that TOB1 may control IRF3 activity and antiviral immune response. In the present study, we determined that viral infection markedly induced the interaction of TOB1 with IRF3, which is bound to the promoter region of *Ifnb1* in macrophages. TOB1 thereby inhibited *Ifnb1* transcription by disrupting IRF3 binding and recruiting histone deacetylase 8 (HDAC8) to the *Ifnb1* promoter region. Consequently, TOB1 attenuated IRF3-directed IFN-β expression in virus-infected macrophages. *Tob1* deficiency enhanced antiviral response and suppressed viral replication in vivo. Therefore, we identified TOB1 as an inhibitor of host antiviral innate immune response and revealed a mechanism of viral immune escape.

## Results

### Viral infection induced an association between TOB1 and IRF3

**Fig. 1:**
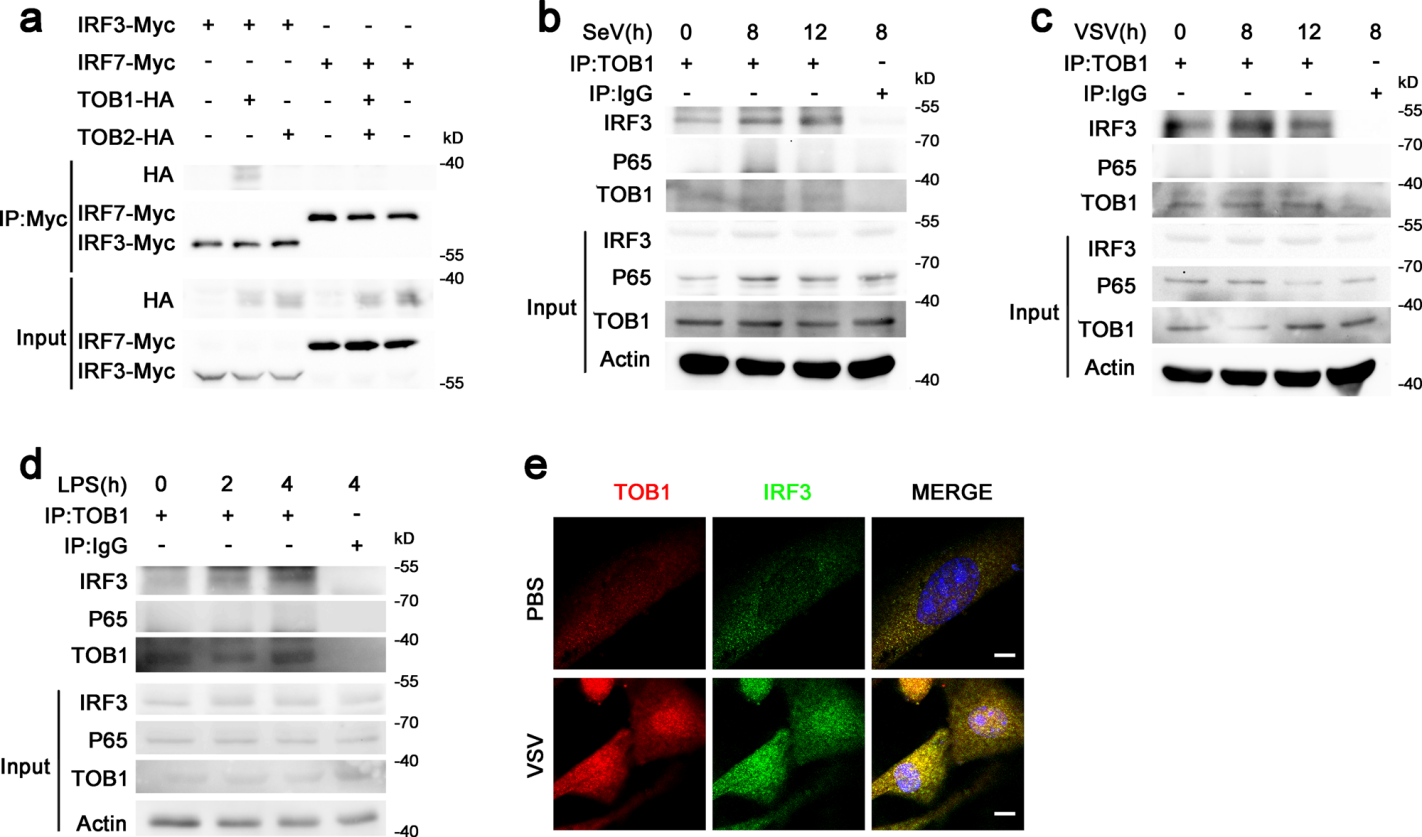
Viral infection induces TOB1-IRF3 interaction. **a** Lysates from HEK293T cells transiently transfected with indicated plasmids were subjected to immunoprecipitation with anti-Myc antibody followed by western blot analysis. **b**–**d** Lysates from mouse peritoneal macrophages (PMs) stimulated with SeV, VSV, or LPS for the indicated time periods were subjected to immunoprecipitation with anti-TOB1 antibody followed by western blot analysis with anti-IRF3 or anti-p65 antibody. Proteins in the whole-cell lysate were used as the positive control (Input). **e** Immunofluorescence analysis of the colocalization of TOB1(red), IRF3 (green), and nucleus(blue) in MEFs stimulated with VSV. Scale bar, 10 μm. Data are shown as a representative result from three independent experiments.

We examined the association between TOB1 and IRF3 to investigate whether TOB1 controls IRF3 activity. The Myc-tagged IRF3 plasmid and HA-tagged TOB1 or TOB2 plasmids were co-transfected into HEK293T cells. TOB1 co-precipitated with IRF3 but not IRF7 (Fig. 1a). However, TOB2 did not co-precipitate with IRF3 (Fig. 1a). Endogenous interactions were examined during viral infection in mouse primary peritoneal macrophages (PMs). As shown in Fig. 1b–d, TOB1 interacted with IRF3 following infection with Sendai virus (SeV, an ssRNA virus recognized by RIG-I), vesicular stomatitis virus (VSV, another ssRNA virus recognized by RIG-I), or lipopolysaccharide (LPS; a Toll-like receptor 4 ligand). However, TOB1 and p65 did not interact (Fig. 1b–d). Furthermore, immunofluorescence assays showed that TOB1 interacted with IRF3 in the cytoplasm of resting mouse embryonic fibroblasts (MEFs), and viral infection further enhanced the interaction and promoted TOB1 and IRF3 translocation to the nucleus (Fig. 1e). Collectively, these results indicate that TOB1 binds to IRF3 and is potentially involved in the regulation of antiviral responses.

### *Tob1* deficiency enhanced IFN-β expression and antiviral immune responses

**Fig. 2:**
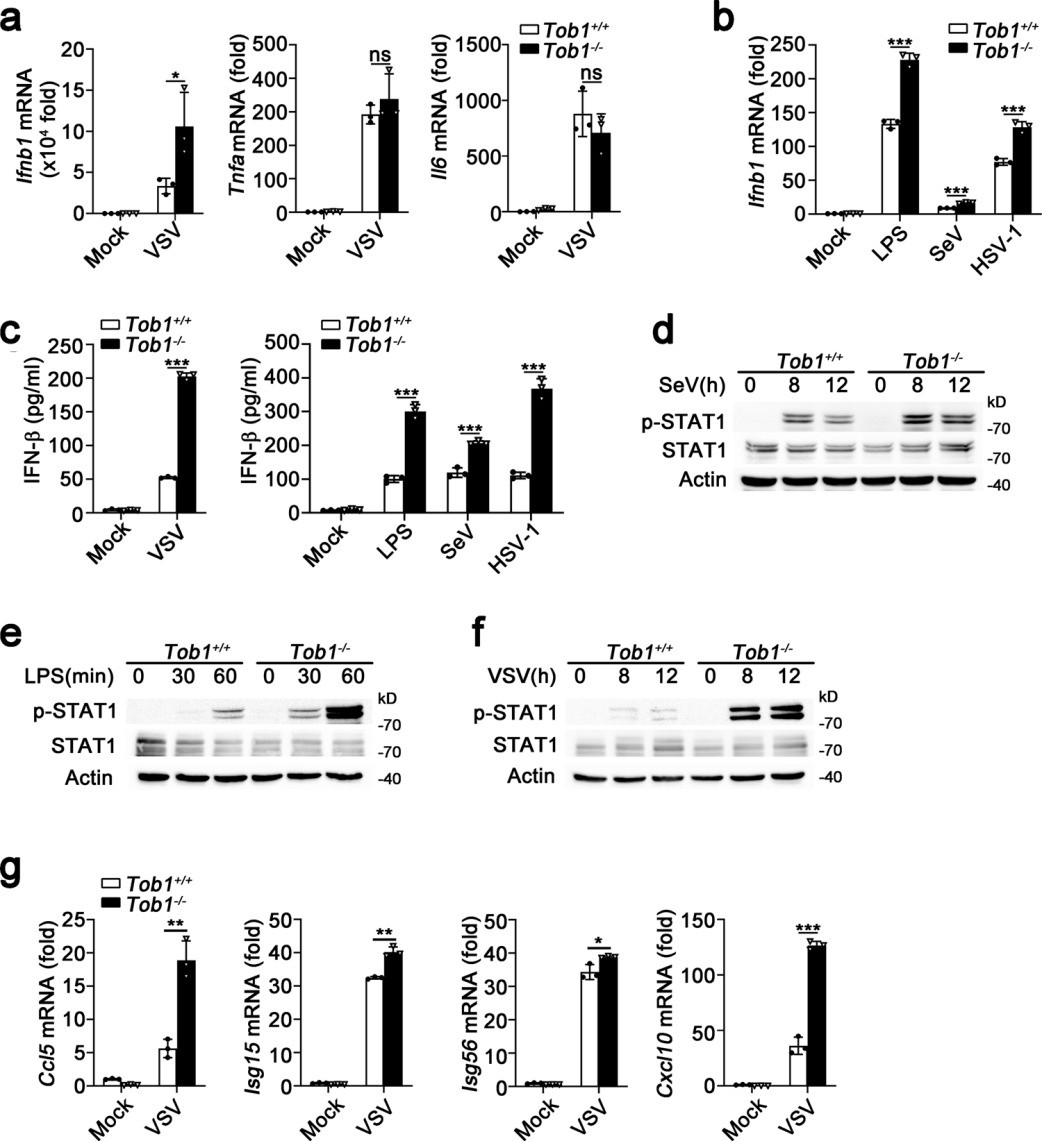
*Tob1* deficiency enhances IFN-β expression. **a** Quantitative real-time RT-PCR analysis of IFN-β, TNF-α, and IL-6 mRNA expression in peritoneal macrophages (PMs) isolated from *Tob1*^+/+^ or *Tob1*^−/−^ mice and then infected with VSV. **b** Quantitative real-time RT-PCR analysis of IFN-β mRNA expression in PMs isolated from *Tob1*^+/+^ or *Tob1*^−/−^ mice and then exposed to lipopolysaccharide (LPS) or infected with SeV or HSV-1. **c** Enzyme-linked immunosorbent assay (ELISA) of IFN-β secretion in the supernatants of PMs from *Tob1*^+/+^ or *Tob1*^−/−^ mice after treatment as indicated. **d**–**f** Western blot analysis of STAT1 phosphorylation in PMs isolated from *Tob1*^+/+^ or *Tob1*^−/−^ mice and then exposed to SeV (**d**), LPS (**e**), or VSV (**f**). **g** Quantitative real-time RT-PCR analysis of *Ccl5, Isg15, Isg56*, and *Cxcl10* mRNA expression PMs isolated from *Tob1*^+/+^ or *Tob1*^−/−^ mice and then infected with VSV. All data are shown as the means ± SD. Significance was determined by unpaired two-tailed Student’s *t*-test: *p < 0.05, **p < 0.01, ***p < 0.001, ns, *p* > 0.05. Data are shown as a representative result from three independent experiments. Data are shown as a representative result from three independent experiments.

Next, we examined the effects of TOB1 on the viral infection-induced innate immune response. *Tob1* deficiency markedly promoted the induction of IFN-β mRNA expression following VSV infection, with no effect on the expression of proinflammatory cytokines, including tumor necrosis factor-α (TNF-α) and IL-6 (Fig. 2a and Supplementary Fig. 1a). Furthermore, the IFN-β mRNA expression induced by LPS, SeV, and herpes simplex virus 1 (HSV-1, a DNA virus recognized by cGAS) was greatly enhanced by *Tob1* deficiency (Fig. 2b). Consistently, *Tob1* deficiency significantly enhanced VSV-, LPS-, SeV-, and HSV-1 induced IFN-β secretion (Fig. 2c). Collectively, these data indicate that TOB1 attenuates Toll-like receptor 4-, RIG-I-, and cGAS-triggered IFN-β expression and regulates the innate antiviral immune response.

IFN-β induces ISG expression and suppresses viral replication through the JAK-STAT pathway^3^. To further investigate the function of TOB1 in antiviral responses and viral elimination, we examined STAT1 activation. *Tob1* deficiency promoted SeV-, LPS-, and VSV-induced STAT1 phosphorylation (Fig. 2d–f). Consistently, VSV-induced expression of C-X-C motif chemokine 10 (*Cxcl10*) and ISGs, including *Ccl5*, *Isg15*, and *Isg56*, was enhanced by *Tob1* deficiency (Fig. 2g). However, *Tob1* deficiency did not affect IFN-β-induced ISG expression or STAT1 phosphorylation (Supplementary Fig. 1b–e). These results indicate that TOB1 inhibits the IFN response upstream of IFN-β production.

### *Tob1* deficiency decreased VSV replication in PMs

**Fig. 3:**
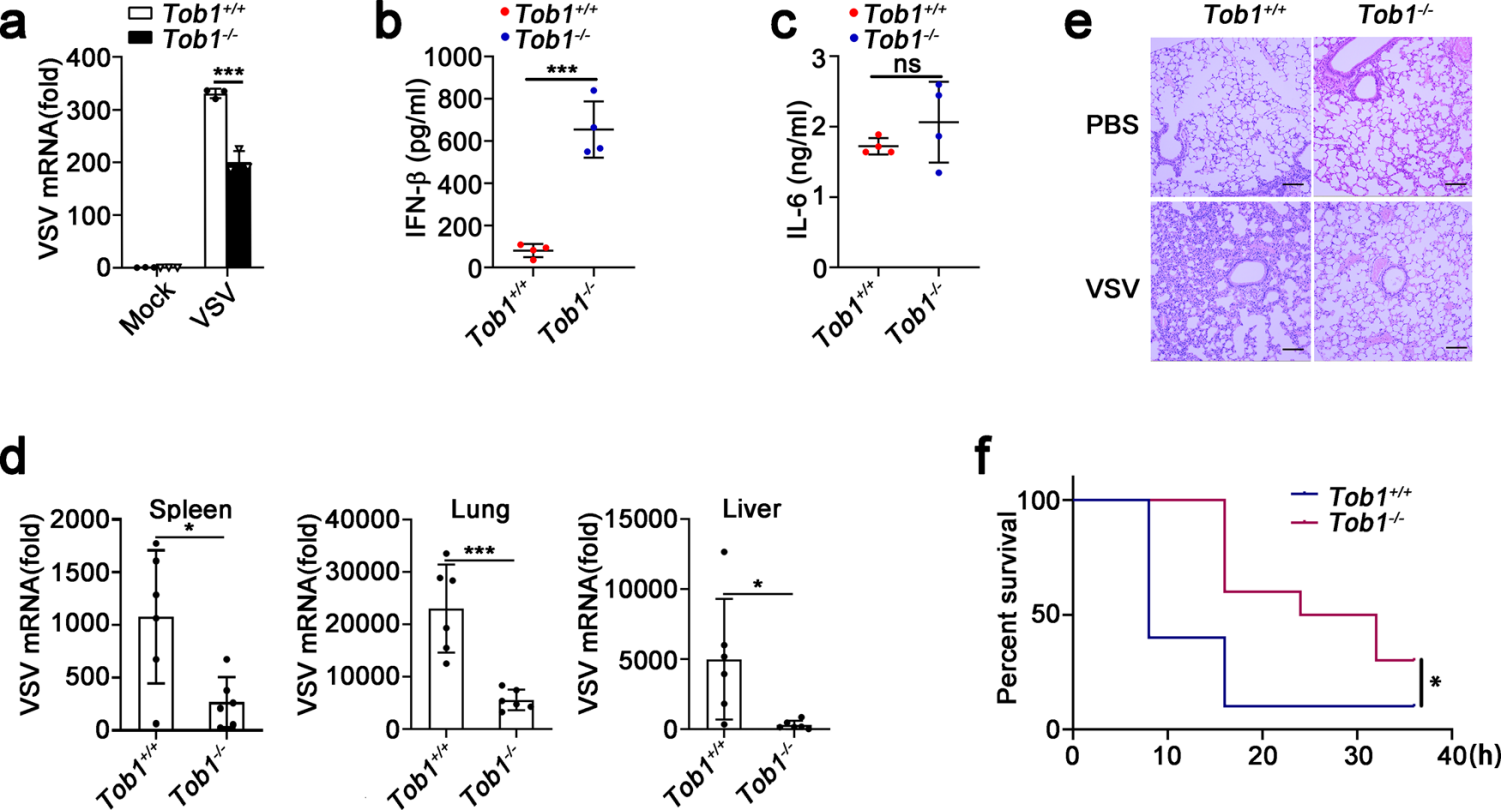
TOB1 deficiency enhance innate immune responses against VSV in vivo. **a** Quantitative real-time RT-PCR analysis of VSV mRNA expression in peritoneal macrophages (PMs) isolated from *Tob1*^+/+^ or *Tob1*^−/−^ mice and then infected with VSV for 8 h. **b**, **c** *Tob1*^+/+^ or *Tob1*^−/−^ mice (*n* = 4) were infected with VSV (4 × 10^7^ pfu/mouse) for 8 h. ELISA of IFN-β (**b**) and IL-6 (**c**) in serum. **d** *Tob1*^+/+^ or *Tob1*^−/−^ mice (*n* = 6) were infected with VSV (4 × 10^7^ pfu/mouse) for 12 h. Quantitative real-time RT-PCR analysis of VSV mRNA in spleen, lung, and liver. **e** Hematoxylin and eosin staining of lung tissue sections with VSV (4 × 10^7^ pfu/mouse) infection for 18 h (PBS, *n* = 3 and VSV, *n* = 3 per condition). Scale bar, 100 μM. **f** Kaplan–Meier method was used to evaluate the survival curves (*n* = 10 per condition). All data are shown as the means ± SD. Significance was determined by unpaired two-tailed Student’s *t*-test: *p < 0.05, **p < 0.01, ***p < 0.001, ns, *p* > 0.05. Data are shown as a representative result from three independent experiments.

Consistent with their inhibitory roles in IFN-β and ISG expression, *Tob1* deficiency inhibited VSV replication in PMs (Fig. 3a). Next, we investigated the physiological and pathological relevance of TOB1 in the context of viral infection in vivo. Higher IFN-β production was observed in the sera of VSV-infected *Tob1*^−/−^ mice than in those of their wild-type littermates (Fig. 3b). However, no difference in IL-6 secretion was observed between the sera of VSV-infected *Tob1*^+/+^ and *Tob1*^−/−^ mice (Fig. 3c). Concordantly, VSV replication was lower in the spleen, lungs, and liver of *Tob1*^−/−^ mice than in their wild-type littermates (Fig. 3d). Furthermore, a lower infiltration of immune cells was observed in the lungs of *Tob1*-deficient mice than in control wild-type mice after VSV infection (Fig. 3e), and *Tob1*-deficient mice were significantly more resistant to VSV-induced lethality than wild-type mice (Fig. 3f). These observations indicate that TOB1 suppresses IFN-β production and antiviral immune response.

### TOB1 inhibited IRF3 binding to *Ifnb1* promoter region

**Fig. 4:**
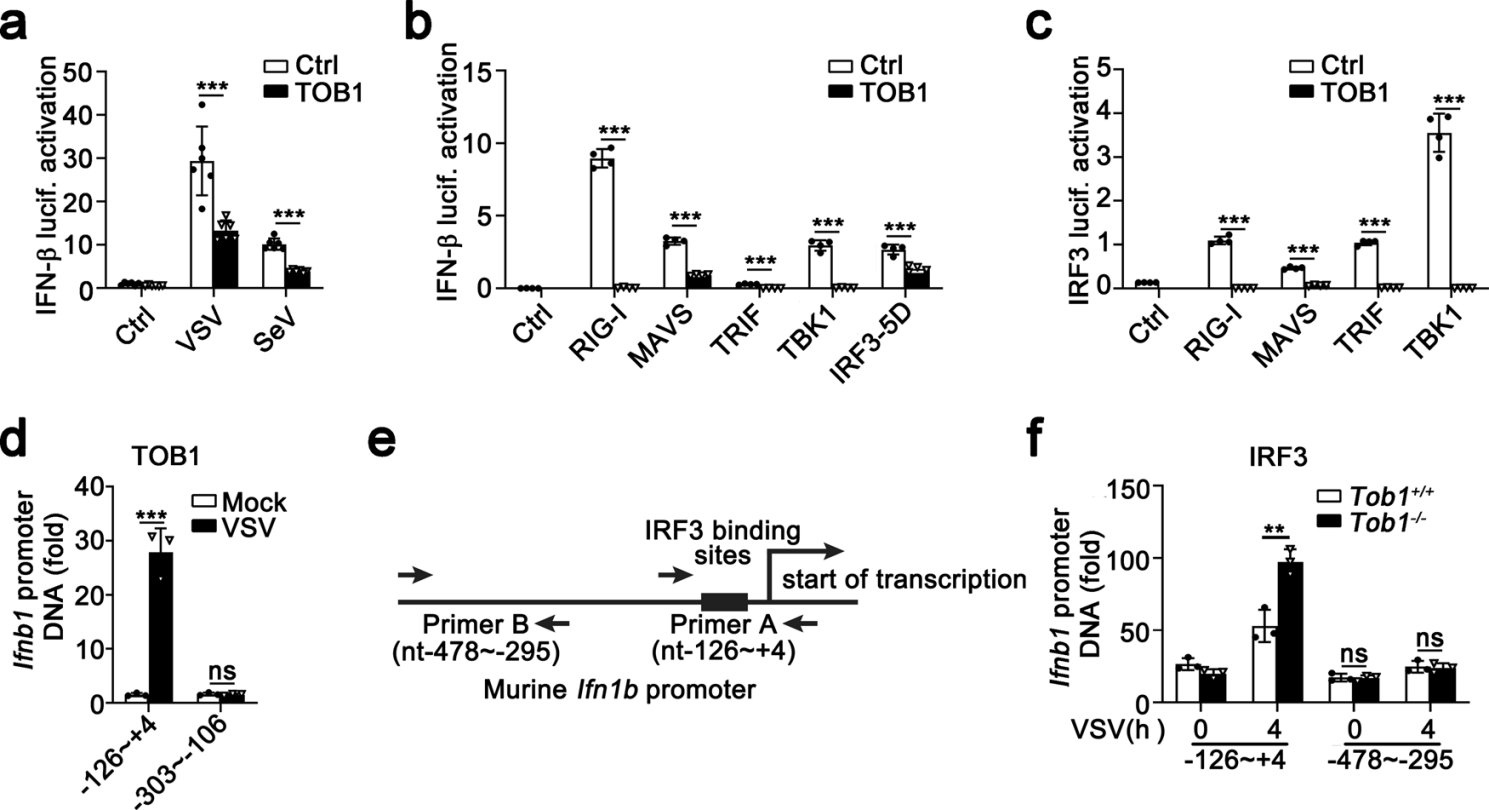
TOB1 inhibits IRF3 binding to *Ifnb1* promoter. **a** Luciferase activity analysis of HEK293T cells transiently transfected with IFN-β reporter plasmid together with TOB1 expression plasmid or empty vector(ctrl) after being infected with VSV or SeV. **b**, **c** Luciferase activity analysis of HEK293T cells transiently transfected with IFN-β (**b**) or IRF3 (**c**) reporter plasmid and adaptor plasmids as indicated, together with TOB1 expression plasmid or empty vector(ctrl). **d** ChIP-qPCR analysis of TOB1 binding at *Ifnb1* loci in VSV-infected mouse primary peritoneal macrophages (PMs). **e** IRF3-binding site in the murine *Ifnb1* promoter. Filled arrows, primers used for ChIP-qPCR assay. **f** *Tob1*^+/+^ or *Tob1*^−/−^ mouse PMs were infected with VSV, and then prepared for ChIP-qPCR assay performed using an anti-IRF3 antibody. All data are shown as the means ± SD. Significance was determined by unpaired two-tailed Student’s *t*-test: **p < 0.01; ***p < 0.001. Data are shown as a representative result from three independent experiments. (*n* = 4 in **a**–**c**; *n* = 3 in **d** and **f**).

To identify the molecular targets of TOB1 in IFN-β signaling, the effects of TOB1 on IFN-β promoter activation, mediated by viruses or various adaptors, were examined. VSV-, SeV, RIG-I-, MAVS-, TRIF-, and TBK1-induced IFN-β promoter activation was significantly attenuated by TOB1 overexpression (Fig. 4a, b), suggesting that TOB1 targets downstream of TBK1. We investigated the role of TOB1 in IRF3-triggered signal transduction. The IFN-β promoter was strongly activated in the IRF3 5D mutant, in which residues at positions 396, 398, 402, 404, and 405 were replaced by the phosphomimetic aspartate amino acid^14^. TOB1 attenuated IRF3 5D-induced IFN-β promoter activation (Fig. 4b), indicating that TOB1 targeted IRF3. To further clarify the mechanism by which TOB1 inhibits the IRF3-dependent pathway, we examined the effect of TOB1 on IRF3 reporter activation. The IRF3 reporter contains two plasmids^15^. One was a fusion expression plasmid in which IRF3 was fused to the GAL4-DNA-binding domain. The second is a luciferase reporter with a promoter fragment that allows GAL4 binding. Therefore, activation of IRF3 allows IRF3-GAL4 binding and drives the expression of luciferase^15^. As shown in Fig. 4c, TOB1 significantly inhibited RIG-I-, MAVS-, TRIF-, and TBK-induced IRF3 luciferase activation. Next, we performed a chromatin immunoprecipitation (ChIP) assay, which showed that TOB1 could bind to the *Ifnb1* promoter (nt −126 to +4) following VSV infection (Fig. 4d). IRF3 also bound to the *Ifnb1* promoter (nt −126 to +4), and *Tob1* deficiency enhanced IRF3 binding to the *Ifnb1* promoter (nt −126 to +4; Fig. 4e, f). We further confirmed whether IFN-α and IFN-γ are regulated by TOB1, and the results showed that *Tob1* deficiency greatly promoted VSV-induced *Ifna4* mRNA (Supplementary Fig. 2a) expression, but not *Ifng* in PMs (Supplementary Fig. 2b). A possible reason for the similar effect of *Tob1*-deficient cells on *Ifnb1* and *Ifna4* is that the mouse *Ifna4* promoter region has a virus-responsive element (VRE-A4) containing IRF3-binding sites and can activate *Ifna4* transcription. Taken together, these data indicate that TOB1 suppresses IRF3 binding to the *Ifnb1* promoter.

### TOB1 recruited HDAC8 and inhibits IFN-β expression

**Fig. 5:**
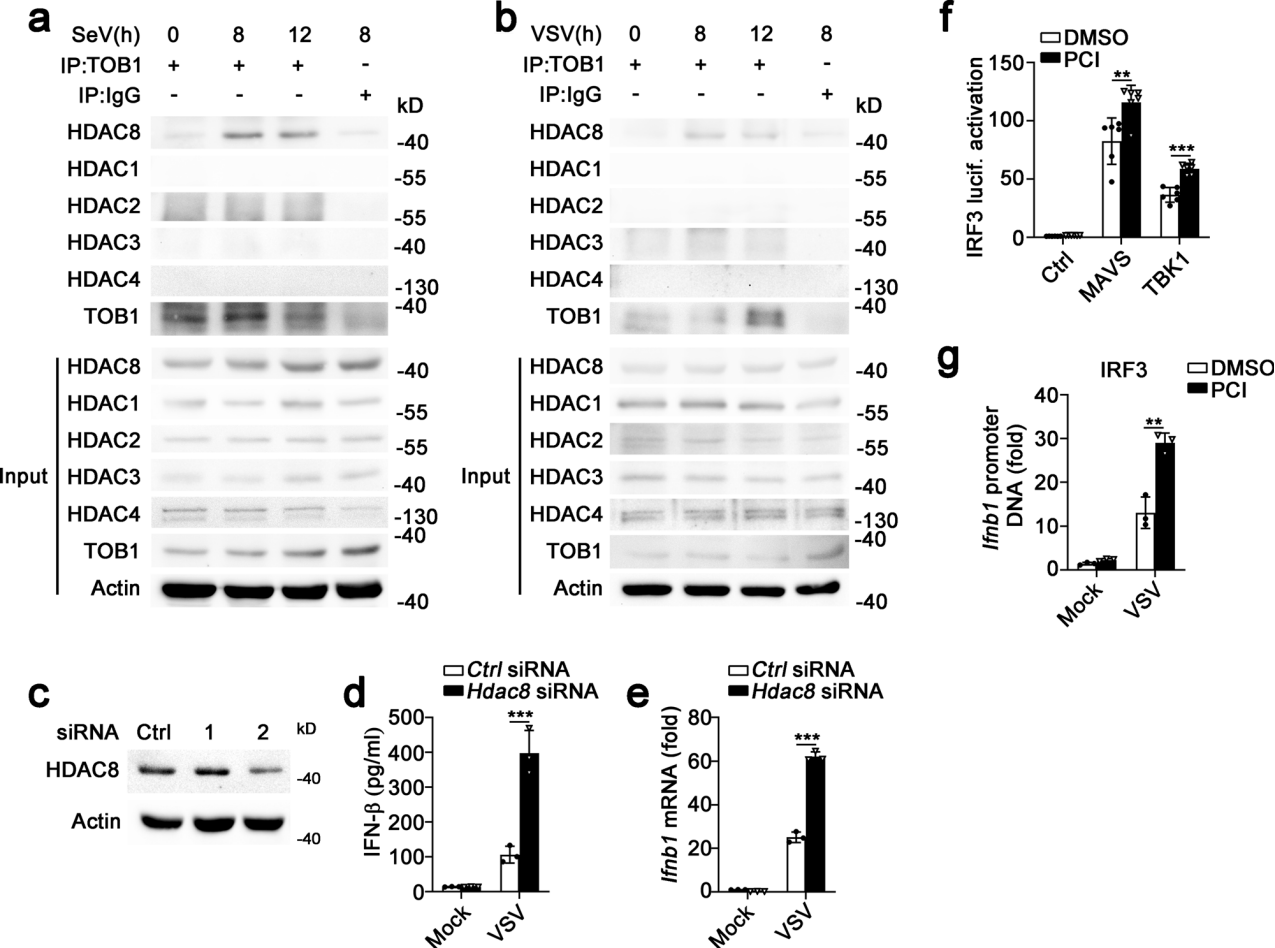
TOB1 recruits HDAC8 to suppress IFN-β expression. **a**, **b** Lysates from mouse PMs infected with SeV (**a**) or VSV (**b**) for the indicated time periods were subjected to immunoprecipitation with anti-TOB1 antibody followed by western blot analysis with indicated antibodies. Proteins in whole-cell lysate were used as positive controls (Input). **c** Immunoblot analysis of HDAC8 expression in mouse primary peritoneal macrophages (PMs) transfected with Ctrl siRNA, HDAC8 siRNA 1 or 2 for 48 h. **d**, **e** ELISA and RT-PCR analysis of IFN-β secretion (**d**) and transcription (**e**) in the supernatants of PMs with Ctrl siRNA or *Hdac8* siRNA 2, and then infected with VSV. **f** Luciferase activity analysis of HEK293T cells transiently transfected with IFN-β reporter plasmid and adaptor plasmids as indicated, followed by treatment with dimethyl sulfoxide (DMSO) or PCI-34051 (5 μM). **g** DMSO or PCI-34501 pretreated mouse primary peritoneal macrophages (PMs) were injected with VSV, and then prepared for ChIP-qPCR assay using anti-IRF3 antibody. All data are shown as the means ± SD. Significance was determined by unpaired two-tailed Student’s *t-*test: **p < 0.01. ***p < 0.001. Data are shown as a representative result from three independent experiments.

**Fig. 6:**
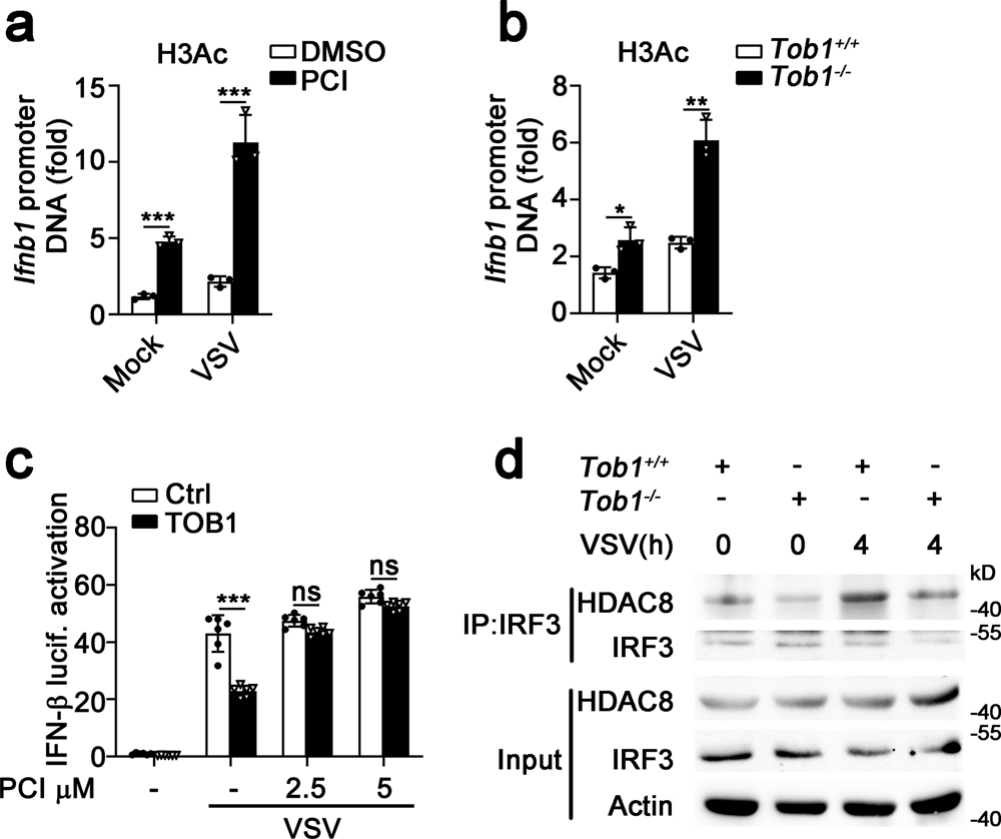
TOB1 enhances deacetylation of *Ifnb1* promoter by recruiting HDAC8. **a** DMSO or PCI-34501 pretreated mouse primary peritoneal macrophages (PMs) were injected with VSV, and then prepared for ChIP-qPCR assay using anti-H3Ac antibody. **b** *Tob1*^+/+^ or *Tob1*^−/−^ mouse PMs were injected with VSV, and then prepared for ChIP-qPCR assay performed using an anti-H3Ac antibody. **c** Luciferase activity analysis of HEK293T cells transiently transfected with IFN-β reporter plasmid, followed by treatment with DMSO or PCI-34051 (5 μM), and then infected with VSV. **d** Lysates from *Tob1*^+/+^ or *Tob1*^−/−^ PMs infected with VSV were subjected to immunoprecipitation with anti-IRF3 antibody followed by western blot analysis with anti-IRF3 or anti-HDAC8 antibody. Proteins in whole-cell lysate were used as positive controls (Input). All data are shown as the means ± SD. Significance was determined by unpaired two-tailed Student’s *t*-test: **p < 0.01. ***p < 0.001. Data are shown as a representative result from three independent experiments.

Histone acetylation is required for gene transcription, whereas histone deacetylation suppresses gene transcription^16^. As TOB1 recruits HDACs to the promoter regions of its target genes^17^, and class I HDACs (including HDAC1, 2, 3, and 8) play inhibitory roles in innate immunity^16,18^, we investigated whether TOB1 recruits class I HDACs to the *Ifnb1* promoter to suppress its transcription. As shown in Fig. 5a, b, TOB1 interacted with HDAC8 following SeV or VSV infection but did not interact with HDAC1, HDAC2, HDAC3, and HDAC4 in macrophages. To confirm the intrinsic role of HDAC8, small interfering RNA (siRNA) knockdown experiments were performed, and siRNAs targeting mouse *Hdac8* were synthesized to suppress endogenous HDAC8 expression (Fig. 5c). *Hdac8* knockdown substantially enhanced the VSV-induced IFN-β secretion and transcription (Fig. 5d, e). Furthermore, PCI-34051 treatment (a selective HDAC8 inhibitor) significantly enhanced both MAVS- and TBK-induced IRF3 luciferase activation and the binding rate between IRF3 and the *Ifnb1* promoter (nt −126 to +4; Fig. 5f, g). PCI-34051 treatment and *Tob1* deficiency increased the acetylation level of histone H3 at the *Ifnb1* promoter following VSV infection (Fig. 6a, b). In addition, the inhibitory effects of TOB1 on IFN-β activation induced by VSV infection were completely blocked in PCI-34051-treated HEK293T cells (Fig. 6c). *Tob1* deficiency inhibited the interaction between HDAC8 and IRF3 in macrophages during VSV infection (Fig. 6d). These observations indicate that TOB1 recruits HDAC8 to the *Ifnb1* promoter to inhibit acetylation of this promoter in response to viral infection and suppress IFN-β expression.

## Discussion

Emerging viral diseases pose a significant threat to human health and are among the most important public health challenges. A better understanding of the crosstalk between viruses and the immune system may facilitate the development of effective preventive and treatment measures. IRF3 is a key transcription factor required for IFN-β transcription and plays a fundamental role in the initiation of antiviral innate immune responses^19^. Viruses can block innate immunity and achieve immune escape by suppressing IRF3 activity. However, excessive IRF3 activation and type I IFN production can lead to the development of various autoimmune diseases^20^. Therefore, IRF3 activity should be tightly controlled to facilitate viral eradication and maintain host immune homeostasis. The IRF3-directed IFN response can be regulated in various ways, such as through post-translational modifications and the modulation of IRF3 transcriptional activity. Post-translational modifications, including phosphorylation, ubiquitination, and acetylation, control IRF3 activity and affect its binding to the *Ifnb1* promoter^21–23^. Class I HDACs, such as HDAC1 and HDAC8, control the IFN response by attenuating *Ifnb1* transcription^16,18^. For example, retinoblastoma protein (Rb) selectively binds to the *Ifnb1* enhancer region by interacting with c-Jun and recruiting HDAC1 and HDAC8, resulting in the suppression of *Ifnb1* transcription^18^. TET3 binds to the *Ifnb1* promoter and recruits HDAC1 to suppress *Ifnb1* transcription^24^. We found that viral infection-induced TOB1 interacted with IRF3 and bound to the promoter region of *Ifnb1*, leading to recruitment of HDAC8 and suppression of IFN-β production (Supplementary Fig. 3). Although viral infections lead to IFN responses in the host, which in turn activate an antiviral defense program to restrict viral replication, many viruses use host proteins to evolve strategies to escape innate immune surveillance, such as the alteration in STAT post-translational modifications and disturbing the formation of IFN-β transcription complexes^25^. *Tob1*-deficient mice secreted higher levels of IFN-β and showed suppressed viral replication compared with wild-type mice. Thus, our results reveal a mechanism by which viruses circumvent the IRF3-directed IFN response.

TOB1, a transcriptional co-repressor, suppresses the expression of several genes. Recently, TOB1 has been implicated in several autoimmune diseases, including multiple sclerosis and experimental autoimmune encephalomyelitis (EAE). *Tob1* deficiency (or downregulation) in patients with clinically isolated syndromes at risk of conversion to clinically definite multiple sclerosis may contribute to the differentiation and proliferation of proinflammatory T-cells and central nervous system autoimmunity^11^. Given the important roles of type I IFNs in multiple autoimmune disorders, TOB1 may play an essential role in the development of autoimmune diseases caused by aberrant IFN responses.

In this study, we demonstrated that TOB1, which is induced during viral infection, inhibited IRF3-directed IFN responses and antiviral immunity. Our results identified a negative feedback regulator (TOB1-HDAC8) that terminated IRF3-directed antiviral response. Therefore, TOB1 is a potential target for preventing and treating diseases caused by aberrant IRF3 responses.

## Methods

### Mice

*Tob1*^−/−^ mice (B6;129S4-*Tob1*^tm1Tya/J^, Stock No:023346; Yoshida et al., 2000) were purchased from Jackson Laboratory. C57BL/6 mice were obtained from the Vital River Laboratory Animal Technology Co. (Beijing, China). *Tob1*^−/−^ mice were crossed with C57BL/6 mice for at least two generations, and experiments were performed on littermate-controlled, co-housed mice. All animal experiments were performed in accordance with the National Institute of Health Guide for the Care and Use of Laboratory Animals, after obtaining approval from the Scientific Investigation Board of the School of Basic Medical Science, Shandong University, Jinan, Shandong Province, China.

### Cells

Mice were administered 3% Brewer’s thioglycollate via intraperitoneal injection to obtain mouse primary peritoneal macrophages (PMs). Peritoneal exudate cells were harvested 3 days later and were cultured for 2 h, after which the nonadherent cells were removed. The remaining adherent monolayer cells were used as peritoneal macrophages^26–28^. Human embryonic kidney (HEK293T) cells were obtained from the American Type Culture Collection (Manassas, VA, USA) and cultured in endotoxin-free Dulbecco’s modified Eagle’s medium containing 10% (v/v) fetal bovine serum (Invitrogen, Carlsbad, CA, USA).

### Antibodies and reagents

Anti-TOB1 (14915-1-AP, 1:500) antibody was purchased from Proteintech (Rosemont, IL, USA). Anti-HDAC8 (ab187139, 1:1000) antibody was purchased from Abcam (Cambridge, UK). Anti-IRF3 (4302,1:1000), anti-STAT1 (9172,1:1000), anti-p-STAT1 (9167,1:1000), anti-p65 (3031 S,1:1000), anti-HDAC1 (5356,1:1000), anti-HDAC2 (5113,1:1000), anti-HDAC3 (3949,1:1000), and anti-HDAC4 (7628,1:1000) antibodies were purchased from Cell Signaling Technology (Danvers, MA, USA). Anti-acetyl-H3 (06-599, 1:50), anti-HA (H3663,1:1000), and anti-Myc (M4439,1:1000) antibodies were purchased from Sigma–Aldrich (St. Louis, MO, USA). Anti-β-actin (sc-81178,1:1000) and protein G agarose (sc-2002,1:100) were used for immunoprecipitation, and horseradish peroxidase-conjugated secondary antibodies were purchased from Santa Cruz Biotechnology (Dallas, TX, USA). Alexa Fluor 633 (A-21071, 1 µg/mL) and Alexa Fluor 488 (A-11059,1 µg/mL) were purchased from Thermo Fisher Scientific (Waltham, MA, USA). Sendai virus (SeV) was purchased from the China Center for Type Culture Collection (Wuhan University, China), and a multiplicity of infection of 1 was used. VSV-GFP and HSV-1 were gifts from Dr. Xuetao Cao (Second Military Medical University). LPS (*Escherichia coli* 055:B5) was purchased from Sigma–Aldrich. The concentration of LPS used was 100 ng/mL. PCI-34051 (s2012) was obtained from Selleck Chemicals (Houston, TX, USA).

### RNA interference

For transient silencing, siRNA duplexes were transfected into PMs using INTERFERin reagent (Polyplus-transfection, Illkirch-Graffenstaden, France), according to the manufacturer’s protocol. The target sequences for transient silencing of mouse *Hdac8* were 5′-UCCAGACUCCAUAGAAUAUTT AUAUUCUAUGGAGUCUGGATT-3′ (siRNA 1), and 5′-GCAGCUAUAGGAGGAGGUATTUACCUCCUCCUAUAGCUGCTT-3′ (siRNA 2) and the control sequence was 5′- UUCUCCGAACGUGUCACGU-3′.

### Plasmids and transfection

TOB1 and TOB2 expression plasmids were constructed using PCR-based amplification of cDNA from THP-1 cells and then cloned into the pFLAG-CMV-2 eukaryotic expression vector (Sigma–Aldrich). The IFN-β and IRF3 reporter plasmids and expression plasmids for RIG-I, MAVS, TRIF, TBK1, and IRF3 5D have been described previously^26–28^. All the constructs were confirmed by DNA sequencing. Lipofectamine^TM^ 2000 reagent (Invitrogen) was used to transiently transfect the plasmids into HEK293T cells, according to the manufacturer’s instructions.

### Enzyme-linked immunosorbent assay

The IFN-β concentration was measured using enzyme-linked immunosorbent assay (ELISA) kits (BioLegend, San Diego, CA, USA). Concentrations of TNF-α and IL-6 were measured using ELISA kits (Dakewe Biotech Company Ltd., Shenzhen, China).

### RNA quantification

Total RNA was extracted using TRIzol reagent according to the manufacturer’s instructions (Invitrogen). A LightCycler (ABI PRISM^®^ 7000, Applied Biosystems, Foster City, CA, USA) and SYBR RT-PCR kit (Takara, Shiga, Japan) were used for the quantitative real-time RT-PCR analysis. The specific primers used for the RT-PCR assays were 5′-ATGAGTGGTGGTTGCAGGC-3′, 5′-TGACCTTTCAAATGCAGTAGATTCA-3′ for *Ifnb1*, 5′-ACGGCGTACTTCCAGATGG-3′ and 5′-CTCGGTTCAAGATCCAGGT-3′ for VSV, 5′-ATGAACGCTACACACTGCATC-3′ and 5′-CCATCCTTTTGCCAGTTCCTC-3′ for Ifng, 5′-CACCACTCCCTGCTGCTTTG-3′ and 5′-ACACTTGGCGGTTCCTTCG-3′ for mCcl5, 5′-ATCATCCCTGCGAGCCTATCCT-3′ and 5′-GACCTTTTTTGGCTAAACGCTTTC-3′ for mCxcl10, 5′-AGAAGCAGATTGCCCAGAAG-3′ and 5′-TGCGTCAGAAAGACCTCATAGA-3′ for Isg15, 5′-GACTTGTCTGCTACTTGGAATGC-3′ and 5′-TTGGTTGAGGAAGAGAGGGCT-3′ for Ifna4, 5′-TGCTGAGATGGACTGTGAGGAA-3′ and 5′-TCTTGGCGATAGGCTACGACTG-3′ for Isg56, 5′-TCTGAGGCAGAAAGGACCAT-3′ and 5′-GTGGAGGATCCACCTGTTGT-3′ for *Ifnb1* promoter (nt −126 to +4), 5′-TCCAGCAATTGGTGAAACTG-3′ and 5′-GATGGTCCTTTCTGCCTCAG-3′ for *Ifnb1* promoter (nt −303 to −106), 5′-AGAGACCCTCTCCCACCATC-3′ and 5′-ATTGCTGGAGCAAAGGAAGA-3′ for *Ifnb1* promoter (nt −478 to −295), and 5′-TGTTACCAACTGGGACGACA-3′, 5′-CTGGGTCATCTTTTCACGGT-3′ for β-actin. The data were normalized to β-actin expression levels in each sample.

### Immunoprecipitation and western blotting

For immunoprecipitation (IP), whole-cell extracts were lysed in IP buffer containing 1.0% (v/v) Nonidet P40, 50 mM Tris-HCl pH 7.4, 50 mM EDTA, 150 mM NaCl, and a protease inhibitor ‘cocktail’ (Merck, Billerica, MA, USA). After centrifugation for 10 min at 14,000 × *g*, the supernatants were collected and incubated with a protein G Plus-Agarose Immunoprecipitation reagent together with a specific antibody. After 6 h of incubation, the beads were washed five times with IP buffer. The immunoprecipitates were eluted by boiling in 1% (w/v) sodium dodecyl sulfate sample buffer. For western blotting, cells were lysed with M-PER Protein Extraction Reagent (Pierce, Rockford, IL) supplemented with a protease inhibitor ‘cocktail’, and protein concentrations in the extracts were measured using a bicinchoninic acid assay (Pierce, Rockford, IL, USA). Equal amounts of the extracts were separated by sodium dodecyl sulfate-polyacrylamide gel electrophoresis and then transferred onto nitrocellulose membranes for immunoblot analysis.

### Luciferase assay

Luciferase activity was measured using the Dual-Luciferase Reporter Assay System (Promega, Madison, WI, USA), according to the manufacturer’s instructions. Data were normalized for transfection efficiency by subtracting firefly luciferase activity from that of *Renilla* luciferase.

### Chromatin immunoprecipitation

Formaldehyde (37%) was added directly to the culture medium at a final concentration of 1% and incubated for 10 min at 37 °C. Glycine was added to a final concentration of 125 mM to quench crosslinking. A ChIP assay kit (Millipore, Billerica, MA, USA) was used according to the manufacturer’s instructions.

### Viral pathogenesis in mice

Mice (female, 7 weeks old) were intravenously infected with VSV (5 × 10^7^ pfu per mouse) as previously described^26,27^. The sera of the mice were collected for ELISA at 12 h after viral infection. Mice were sacrificed to obtain spleen, lung, and liver tissues for qPCR 12 h after viral infection. Lungs from control or virus-infected mice were dissected, fixed in 10% phosphate-buffered formalin, embedded in paraffin, sectioned, stained with hematoxylin and eosin, and examined by light microscopy for histological changes. For virus infection survival experiments, mice were monitored for survival after viral infection.

### Immunofluorescence

mouse embryo fibroblasts (MEFs) were plated on glass coverslips in 24-well plates and incubated with primary antibodies (TOB1 or IRF3) with permeabilization for 90 min. The cells were washed with phosphate-buffered saline (PBS) twice, and the secondary antibody (Alexa Fluor 633 or Alexa Fluor 488) was added directly to the center of the coverslip and incubated for 30 min. The cells were observed using a Zeiss LSM880 confocal laser microscope.

### Statistics and reproducibility

Comparisons between two groups were performed using the unpaired two-tailed Student’s *t*-test, with a P-value <0.05 considered statistically significant. Survival curves were compared using the Kaplan–Meier survival method.

### Reporting summary

Further information on research design is available in the Nature Research Reporting Summary linked to this article.

## Supplementary information

Supplemental Material

Description of Additional Supplementary Files

Supplementary Data 1

Reporting Summary

## Data Availability

The data supporting the findings of this study are available upon request from the corresponding author.
