# Supplementary material for: TOB1 attenuates IRF3-directed antiviral responses by recruiting HDAC8 to specifically suppress IFN-β expression
Source: Commun Biol. 2022 Sep 9;5:943. doi: 10.1038/s42003-022-03911-x (PMC9463440; doi:10.1038/s42003-022-03911-x)
Supplement: Supplementary file 1 — Supplemental Material [file 42003_2022_3911_MOESM1_ESM.pdf]

Supplementary Figure 1. *Tob1* deficiency showed no effect on IFN-β-induced ISGs expression and STAT1 phosphorylation.

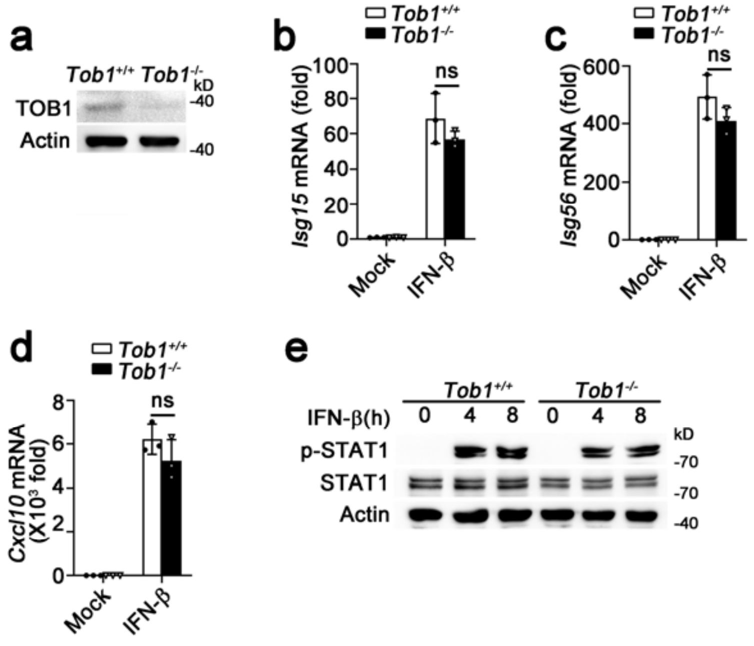

(a) Protein levels of TOB1 in mouse primary peritoneal macrophages(PMs) from *Tob1*$^{+/+}$ or *Tob1*$^{-/-}$ mice. (**b-d**) RT-PCR analysis of IFN-β-induced *Cxcl10* and ISGs expression in PMs from *Tob*$^{1+/+}$ or *Tob1*$^{-/-}$ mice. **(e)** Immunoblot analysis of IFN-β-induced STAT1 phosphorylation in PMs from *Tob*$^{1+/+}$ or *Tob1*$^{-/-}$ mice. All data are shown as the means ± SD. Significance was determined by unpaired two-tailed Student's *t* test. Data are shown as a representative result from three independent experiments.

Supplementary Figure 2. *Tob1*-deficient differently regulates *Ifna4* and *Ifng* mRNA expression.

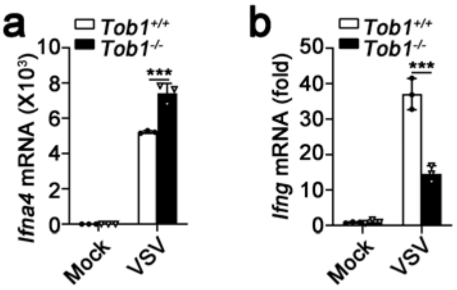

(a,b) Quantitative real-time RT-PCR analysis of *Ifna4* (a) and *Ifng* (b) mRNA expression in mouse primary peritoneal macrophages (PMs) from *Tob*$^{1+/+}$ or *Tob1*$^{-/-}$ mice after infection with VSV. All data are shown as the means ± SD. Significance was determined by unpaired two-tailed Student's t test: ***, p < 0.001. Data are shown as a representative result from three independent experiments.

Supplementary Figure 3. Working model for TOB1 inhibiting IFN-β expression.

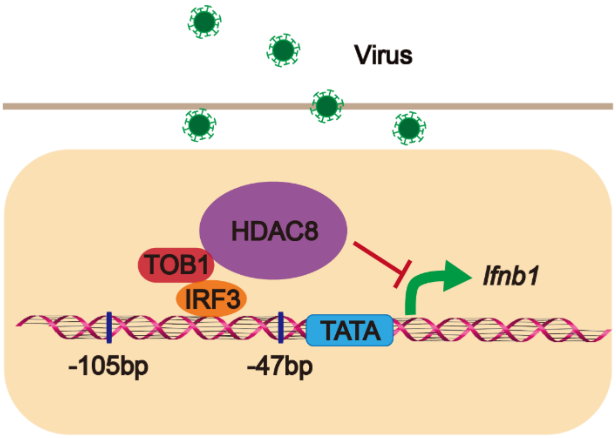

This graphic illustrates the functional model presented for TOB1 during viral infection and how it inhibits IFN-β expression.
