# Supplementary material for: TOB1 attenuates IRF3-directed antiviral responses by recruiting HDAC8 to specifically suppress IFN-β expression
Source: Commun Biol. 2022 Sep 9;5:943. doi: 10.1038/s42003-022-03911-x (PMC9463440; doi:10.1038/s42003-022-03911-x)
Supplement: Supplementary file 4 — Reporting Summary [file 42003_2022_3911_MOESM4_ESM.pdf]

nature portfolio

Corresponding author(s): Mutian Jia; Wei Zhao

Last updated by author(s): Aug 18, 2022

Reporting Summary

Nature Portfolio wishes to improve the reproducibility of the work that we publish. This form provides structure for consistency and transparency in reporting. For further information on Nature Portfolio policies, see our Editorial Policies and the Editorial Policy Checklist.

Statistics

For all statistical analyses, confirm that the following items are present in the figure legend, table legend, main text, or Methods section.

n/a	Confirmed	
☐	☒	The exact sample size (*n*) for each experimental group/condition, given as a discrete number and unit of measurement
☐	☒	A statement on whether measurements were taken from distinct samples or whether the same sample was measured repeatedly
☐	☒	The statistical test(s) used AND whether they are one- or two-sided<br>*Only common tests should be described solely by name; describe more complex techniques in the Methods section.*
☒	☐	A description of all covariates tested
☒	☐	A description of any assumptions or corrections, such as tests of normality and adjustment for multiple comparisons
☐	☒	A full description of the statistical parameters including central tendency (e.g. means) or other basic estimates (e.g. regression coefficient) AND variation (e.g. standard deviation) or associated estimates of uncertainty (e.g. confidence intervals)
☐	☒	For null hypothesis testing, the test statistic (e.g. *F*, *t*, *r*) with confidence intervals, effect sizes, degrees of freedom and *P* value noted<br>*Give P values as exact values whenever suitable.*
☒	☐	For Bayesian analysis, information on the choice of priors and Markov chain Monte Carlo settings
☒	☐	For hierarchical and complex designs, identification of the appropriate level for tests and full reporting of outcomes
☒	☐	Estimates of effect sizes (e.g. Cohen's *d*, Pearson's *r*), indicating how they were calculated

Our web collection on statistics for biologists contains articles on many of the points above.

Software and code

Policy information about availability of computer code

Data collection	Applied Biosystems StepOnePlus (Thermo Fisher Scientific,USA), Amersham Imager 680 (GE Healthcare Life Sciences,USA), GeneGnome XR (Synoptics Ltd. Synoptics Ltd, England), Infinite M200 Pro (Tecan,Switzerland), LSM 780 with Airyscan (Zeiss, Germany).
Data analysis	ImageJ (ImageJ 1.38e, Bethesda, USA), Prism 6(GraphPad,California,USA).

For manuscripts utilizing custom algorithms or software that are central to the research but not yet described in published literature, software must be made available to editors and reviewers. We strongly encourage code deposition in a community repository (e.g. GitHub). See the Nature Portfolio guidelines for submitting code & software for further information.

Data

Policy information about availability of data

All manuscripts must include a data availability statement. This statement should provide the following information, where applicable:

- Accession codes, unique identifiers, or web links for publicly available datasets
- A description of any restrictions on data availability
- For clinical datasets or third party data, please ensure that the statement adheres to our policy

The authors declare that data that support this study are available within the article and its Supplementary Information files or available from the authors upon request.

Human research participants

Policy information about studies involving human research participants and Sex and Gender in Research.

Reporting on sex and gender	*Use the terms sex (biological attribute) and gender (shaped by social and cultural circumstances) carefully in order to avoid confusing both terms. Indicate if findings apply to only one sex or gender; describe whether sex and gender were considered in study design whether sex and/or gender was determined based on self-reporting or assigned and methods used. Provide in the source data disaggregated sex and gender data where this information has been collected, and consent has been obtained for sharing of individual-level data; provide overall numbers in this Reporting Summary. Please state if this information has not been collected. Report sex- and gender-based analyses where performed, justify reasons for lack of sex- and gender-based analysis.*
Population characteristics	*Describe the covariate-relevant population characteristics of the human research participants (e.g. age, genotypic information, past and current diagnosis and treatment categories). If you filled out the behavioural & social sciences study design questions and have nothing to add here, write "See above."*
Recruitment	*Describe how participants were recruited. Outline any potential self-selection bias or other biases that may be present and how these are likely to impact results.*
Ethics oversight	*Identify the organization(s) that approved the study protocol.*

Note that full information on the approval of the study protocol must also be provided in the manuscript.

Field-specific reporting

Please select the one below that is the best fit for your research. If you are not sure, read the appropriate sections before making your selection.

☒ Life sciences ☐ Behavioural & social sciences ☐ Ecological, evolutionary & environmental sciences

For a reference copy of the document with all sections, see nature.com/documents/nr-reporting-summary-flat.pdf

Life sciences study design

All studies must disclose on these points even when the disclosure is negative.

Sample size	Sample size for each experiment is indicated in the legend. No statistical methods were used to predetermine sample sizes. Sample size was chosen based on previous experiments and comparable. The reference of cellular experiments sample size is: H Chung, et al. NLRP3 regulates a non-canonical platform for caspase-8 activation during epithelial cell apoptosis. Cell Death & Differentiation. 23, pages1331–1346(2016).
Data exclusions	No data were excluded from the analyses.
Replication	All experimental findings were reproduced in multiple independent experiments. For experiments using mouse peritoneal macrophages, each independent experiment used mouse peritoneal macrophages isolated from another mouse. For each figure, the number of independent experiments or biological replicates is indicated in the figure legends. Western blot pictures are from a representative experiment and the number of independent repeats is clearly indicated in the figure legends.
Randomization	No statistical methods were used for randomization. For in vitro experiments, mouse peritoneal macrophages were isolated from randomly chosen wild-type or KO mice. For in vivo experiments, wild-type or KO mice were randomly allocated into experimental groups.
Blinding	The investigators were blinded during data collection and analysis where possible, such as RT-PCR, ELISA and confocal microscopy.

Reporting for specific materials, systems and methods

We require information from authors about some types of materials, experimental systems and methods used in many studies. Here, indicate whether each material, system or method listed is relevant to your study. If you are not sure if a list item applies to your research, read the appropriate section before selecting a response.

Materials & experimental systems

n/a	Involved in the study
☐	☒ Antibodies
☐	☒ Eukaryotic cell lines
☒	☐ Palaeontology and archaeology
☐	☒ Animals and other organisms
☒	☐ Clinical data
☒	☐ Dual use research of concern

Methods

n/a	Involved in the study
☒	☐ ChIP-seq
☒	☐ Flow cytometry
☒	☐ MRI-based neuroimaging

Antibodies

Antibodies used

Anti-TOB1 (14915-1-AP) antibody was purchased from Proteintech (Rosemont, IL, USA). Anti-HDAC8 (ab187139) was purchased from Abcam (Cambridge, UK). Anti-IRF3 (4302), anti-STAT1 (9172), anti-p-STAT1 (9167), anti-p65 (3031S), anti-HDAC1 (5356), anti-HDAC2 (5113), anti-HDAC3 (3949), and anti-HDAC4 (7628) were purchased from Cell Signaling Technology (Danvers, MA, USA). Anti-acetyl-H3 (06-599), anti-HA (H3663) and anti-Myc (M4439) antibodies were purchased from Sigma (St. Louis, MO, USA). Anti-β-actin (sc-81178) was Santa Cruz Biotechnology (Dallas, TX, USA). Alexa Fluor 633 (A-21071) and Alexa Fluor 488 (A-11059) were purchased from Thermo Fisher Scientific (Waltham, MA, USA).

Validation

All antibodies were obtained from indicated commercial vendors with ensured quality. All the antibodies used in this study have been validated by the vendors as indicated on the websites. Citations are listed as below:
Anti-TOB1 (14915-1-AP) validate in human for WB: Wang D et al. Phosphorylation of TOB1 at T172 and S320 is critical for gastric cancer proliferation and progression. Am J Transl Res. 2019 Aug 15;11(8):5227-5239.
Anti-HDAC8 (ab187139) validate in human for WB: Zhang R et al. HDAC8-dependent deacetylation of PKM2 directs nuclear localization and glycolysis to promote proliferation in hepatocellular carcinoma. Cell Death Dis. 2020 Dec 5;11(12):1036.
Anti-IRF3 (4302) validate in human for WB:Vail KJ et al. The opportunistic intracellular bacterial pathogen Rhodococcus equi elicits type I interferon by engaging cytosolic DNA sensing in macrophages. PLoS Pathog. 2021 Sep 2;17(9):e1009888.
anti-STAT1 (9172) validate in human for WB: Chikhalya A et al. Human IFIT3 Protein Induces Interferon Signaling and Inhibits Adenovirus Immediate Early Gene Expression. mBio. 2021 Dec 21;12(6):e0282921.
anti-p65 (3031S) validate in human for WB: Perez-Nievas BG et al. Astrocytic C-X-C motif chemokine ligand-1 mediates β-amyloid-induced synaptotoxicity. J Neuroinflammation. 2021 Dec 28;18(1):306.
anti-HDAC1 (5356) and anti-HDAC3 (3949) validate in human for WB: Lo Cascio C et al. Nonredundant, isoform-specific roles of HDAC1 in glioma stem cells. JCI Insight. 2021 Sep 8;6(17):e149232.
anti-HDAC2 (5113) validate in human for WB: Li X et al. The methyltransferase METTL3 negatively regulates nonalcoholic steatohepatitis (NASH) progression. Nat Commun. 2021 Dec 10;12(1):7213.
anti-HDAC4 (7628) validate in human for WB: Guo Y et al. The Ca2+-activated cation channel TRPM4 is a positive regulator of pressure overload-induced cardiac hypertrophy. Elife. 2021 Jun 30;10:e66582.
Anti-acetyl-H3 (06-599): Young AP, Longmore GD. Differences in stability of repressor complexes at promoters underlie distinct roles for Rb family members. Oncogene. 2004 Jan 22;23(3):814-23.
anti-HA (H3663): Park JK et al. Evaluation of Preexisting Anti-Hemagglutinin Stalk Antibody as a Correlate of Protection in a Healthy Volunteer Challenge with Influenza A/H1N1pdm Virus. mBio. 2018 Jan 23;9(1):e02284-17.
anti-Myc (M4439): Kim T et al. Role of MYC-regulated long noncoding RNAs in cell cycle regulation and tumorigenesis. J Natl Cancer Inst. 2015 Feb 6;107(4):dju505.
Anti-β-actin validate in human for WB: Wang B et al. Tubular-specific CDK12 knockout causes a defect in urine concentration due to premature cleavage of the slc12a1 gene. Mol Ther. 2022 May 16:S1525-0016(22)00310-0.
Alexa Fluor 633 (A-21071) for immunofluorescence assays:Schneider MWG, Gibson BA, Otsuka S, Spicer MFD, Petrovic M, Blaukopf C, Langer CCH, Batty P, Nagaraju T, Doolittle LK, Rosen MK, Gerlich DW. A mitotic chromatin phase transition prevents perforation by microtubules. Nature. 2022 Aug 3.
Alexa Fluor 488 (A-11059) for immunofluorescence assays:Lieber CM, Cox RM, Sourimant J, Wolf JD, Juergens K, Phung Q, Saindane MT, Smith MK, Sticher ZM, Kalykhalov AA, Natchus MG, Painter GR, Sakamoto K, Greninger AL, Plemper RK. SARS-CoV-2 VOC type and biological sex affect molnupiravir efficacy in severe COVID-19 dwarf hamster model. Nat Commun. 2022 Jul 29;13(1):4416.

Eukaryotic cell lines

Policy information about cell lines and Sex and Gender in Research

Cell line source(s)	Human embryonic kidney (HEK293T) cells were obtained from American Type Culture Collection (Manassas, VA).
Authentication	None of the cell lines have been authenticated.
Mycoplasma contamination	he cell lines were not tested for mycoplasma contamination.
Commonly misidentified lines (See ICLAC register)	No commonly misidentified cell lines were used.

Animals and other research organisms

Policy information about studies involving animals; ARRIVE guidelines recommended for reporting animal research, and Sex and Gender in Research

Laboratory animals	Tob1−/− mice (B6;129S4-Tob1tm1Tya/J, Stock No: 023346) were purchased from the Jackson Laboratory. For animal experiments with Tob1−/− mice , littermate controls with normal TOB1 expression were used. C57BL/6 mice were from Vital River Laboratory Animal Technology Co. (Beijing, China). Mouse primary peritoneal macrophages were obtained from 4–6 weeks old female mice. Mouse embryonic fibroblasts were generated from 10 weeks female pregnant for 13-14 days mice. For in vivo experiments, 6 weeks old females were used.
Wild animals	Study did not involve wild animals.
Reporting on sex	Both sexes were used in the analysis and sex information was not collected.
Field-collected samples	Study did not involve samples collected from field.
Ethics oversight	All animal experiments were undertaken in accordance with the National Institute of Health Guide for the Care and Use of Laboratory Animals, with the approval of the Scientific Investigation Board of Medical School of Shandong University (Jinan, Shandong Province, China).

Note that full information on the approval of the study protocol must also be provided in the manuscript.
